# The gastrocnemius and soleus muscles deficits in functional ankle instability: an observational study

**DOI:** 10.1186/s12891-025-09286-4

**Published:** 2025-11-14

**Authors:** Nasr Awad Abdelkader, Mostafa Mohammed Khafagaa, Marihan Zakaria Aziz

**Affiliations:** 1 Department of Physical Therapy for Musculoskeletal Disorder and its Surgery, Faculty of Physical Therapy, Cairo University, Giza, Egypt; 2 Physiotherapy Department, School of Health and Social Work, University of Hertfordshire hosted by GAF ( UH-GAF), Cairo, Egypt

**Keywords:** Balance, Gastrocnemius and soleus muscles strength, Functional ankle instability, Functional performance, Isokinetic dynamometry

## Abstract

**Background:**

Functional ankle instability (FAI) is a common condition affecting active populations, often caused by deficits in postural and neuromuscular control. This study aimed to compare the differences in gastrocnemius and soleus muscles strength (eccentric and concentric) between the affected and non-affected limbs at velocities of 60°/120° seconds and find out the relationship between muscle strength, balance, and functional performance of the affected limb in participants with FAI.

**Methods:**

Thirty-eight participants with unilateral FAI, their ages ranging from 15 to 32 years, with a body mass index between 18.5 and 24.9 kg/m²were recruited. They had a history of at least one unilateral lateral ankle sprain that required immobilization for three days or longer, in addition to at least one recurring sprain within the three-to-six-month period before study participation. The FAI was identified if the total score was 11 or above on the identification of functional ankle instability (IdFAI) questionnaire. Participants were assessed using Cybex isokinetic dynamometry, a single-leg stance (SLS) (Eyes open and closed), a Y-balance test (YBT) and a side hop test (SHT), for concentric and eccentric plantarflexion strength at 60°/s and 120°/s, static and dynamic balance, and functional performance, respectively. Data analysis included t-tests, Pearson correlation coefficients, and multi-regression analysis.

**Results:**

Significant differences were found in eccentric torque at 60°/s between affected and non-affected limbs (*p* = 0.048). Moderate indirect correlations were observed between SHT and all strength measures in the affected limb (*r* = -0.51 to -0.61, *p* < 0.001). The YBT showed moderate to strong direct correlations with strength measures. Multiple regression analysis revealed that strength deficits significantly predicted static balance with eyes closed (R² = 0.417, *p* < 0.05).

**Conclusion:**

The deficit of gastrocnemius and soleus muscles eccentric strength significantly impairs function in participants with FAI, especially in SHT and YBT. Strength deficits significantly predicted a lack of functional performance by 41% in the SLS with eyes closed. Velocity-specific strength assessments are crucial for effective interventions, and rehabilitation should focus on eccentric strengthening exercises to enhance dynamic stability and reduce recurrent instability.

**Trial registration:**

The study was registered to ClinicalTrials.gov with the number NCT06715033(Retrospectively registered on 27/11/2024).

## Introduction

Chronic ankle instability (CAI) is a prevalent and debilitating condition that significantly impacts athletic performance and daily functioning, affecting up to 46% of individuals following an initial ankle sprain, ranging from 9 to 76% [1]. It is either mechanical or functional ankle instability (FAI) [2]. Functional ankle instability is defined as the involuntary ankle movement in a physiologically normal range of motion and is one of the most common permanent conditions after ankle sprains with a prevalence of 40% [3]. It is characterized by deficiencies in strength, neuromuscular control, proprioception, and postural stability [4]. The diagnosis of FAI relies on patient perception of symptoms due to impaired proprioception nerve receptors in the ligaments. Accurate diagnosis requires clinical assessment, as treatment approaches vary. Arthroscopic examination has not revealed morphological abnormalities in the anterior talofibular ligament (ATFL), even with noticeable lateral laxity. Research suggests that FAI may be partly due to micro instability from ATFL insufficiency [5]. Akey factor contributing to FAI is the disruption of agonist-antagonist muscle balance around the ankle joint, particularly between the dorsiflexors and plantar flexors, and between the evertors and invertors. This imbalance compromises dynamic joint stability by altering force production and motor control. Patients with FAI often show significant weakness in the dorsiflexors and evertors, resulting in abnormal dorsiflexion/plantarflexion (D/P) and eversion/inversion (E/I) torque ratios. These altered ratios negatively impact joint stabilization mechanisms, especially during high-demand tasks such as cutting, jumping, or sudden directional changes, which are common in athletic activities. As a result, patients demonstrate increased postural sway and a diminished capacity to recover from external perturbations, both of which contribute to a heightened risk of recurrent ankle injuries [6].

The FAI has an impact on the entire lower limb kinetic chain, not only the ankle joint. Altered biomechanics caused by ankle instability can result in compensatory mechanisms in the knee and hip, potentially causing changed gait patterns and an increased risk of injuries in other joints [7–9].

Many studies on ankle strength deficits focused on peroneal muscle weaknesses due to their anatomical proximity to static ligaments affected by inversion injuries and revealed that the peroneus longus has a substantial role for dynamic stability and preventing ankle inversion [10–18]. Research suggests diminished eccentric plantar flexors or strength in FAI individuals, impacting balance and performance. The gastrocnemius and soleus muscles are essential for ankle stability and dynamic control during locomotion. Ankle plantar flexors strength is crucial for concentric force generation during propulsion and eccentric control when decelerating or resisting external forces [19]. However, it is less known about how the gastrocnemius and soleus muscle strength act in patients with FAI.

However, there is limited evidence assessing both concentric and eccentric contractions of the gastrocnemius and soleus muscles, particularly at various angular velocities that reflect different functional demands.

Insufficient plantar flexors strength may affect performance in tasks that require rapid force production and dynamic stability, causing an increasing risk of recurrent ankle sprains [20]. The understanding of the relationship between gastrocnemius and soleus muscles strength deficits and functional performance in individuals with FAI is limited, hindering the development of effective rehabilitation strategies [21]. Static and dynamic balance performance is crucial for injury prevention and rehabilitation, particularly in FAI patients [22]. The single-leg stance (SLS) is the strongest predictor of treatment outcome, aiding in determining baseline balance abilities and functional limitations [23]. The Y-balance test (YBT) is a useful tool for assessing dynamic balance, predicting ankle instability, and evaluating postural control [24].It can also reveal deficiencies in muscular strength and performance limits, providing a guideline for rehabilitation outcomes [25]. The side hop test (SHT) is a method that assesses mediolateral ankle stability, agility, endurance, and dynamic neuromuscular control, which are often reduced in individuals with FAI. It involves repetitive lateral hops over a 30-cm distance to replicate inversion-eversion challenges, making it a valid and sensitive tool for detecting ankle instability functional deficits [26] However, the relationship between gastrocnemius and soleus muscles muscle strength at various contraction modes and velocities and functional performance outcomes has not been studied.

This study attempted to address these gaps by comprehensively examining gastrocnemius and soleus muscles strength deficits in patients with FAI, using both isokinetic strength measurements and functional performance tests. Thus, this study aimed to compare gastrocnemius and soleus muscles strength (concentric and eccentric) between the affected limb with FAI and the non-affected limb and explore the relationship between the gastrocnemius and soleus muscles strength at different velocities (60°/s and 120°/s) and functional performance outcomes. This research is crucial for guiding focused rehabilitation approaches and identifying specific strength deficits predicting poor functional performance.

## Methods

### Study population

An observational comparative study design compared the affected limbs with FAI to the matched non-affected limbs in the same participants, utilizing both isokinetic strength measurements and functional tests. The study was conducted at Biomechanics’ Lab, Al-Hayah University, Cairo, Egypt between May and October 2024.Participants were recruited from an outpatient clinic at Al-Hayah University.

The study was conducted on thirty-eight participants with unilateral FAI. The included participants’ age range was 15 to 32 years, with a body mass index (BMI) of 18.5–27.9 kg/m², and established criteria by the International Ankle Consortium for identifying individuals with CAI [10]. Eligible participants had their first episode of significant ankle sprain that occurred at least 12 months before study enrollment resulting in instability and impaired physical activity. They also had to report at least two episodes of ankle instability history of the previously injured ankle joint within previous 3 months”. They had at least two incidents of giving way or a sensation of looseness in the same ankle within the past six months. Additionally, participants were required to score 11 or higher on the Arabic version of IdFAI questionnaire [27, 28], with negative anterior drawer and talar tilt tests. Exclusion criteria were a history of fracture in either lower limb requiring realignment; lower limbs previous surgeries, acute injury to other joints of the lower extremity within the last 3 months that impacted joint integrity and function, and bilateral ankle instability [29]. The contralateral limb was screened using the Arabic version of IdFAI questionnaire and clinical history to ensure it did not meet the criteria for instability. Participants who had received any ankle-related physical therapy intervention in any ankle side within the past six weeks prior the study were excluded from the study.

Each participant’s affected limb was included in the study (affected group), while their non-affected limb served as a comparative (non-affected group), allowing for within-subject comparisons. This approach helped to control individual variability in factors such as overall muscle strength, proprioception, and neuromuscular control. The Arabic version of IdFAI questionnaire, which has been validated for identifying FAI participants [30], was employed in ensuring accurate population identification.

### Assessment procedures

**Fig. 1:**
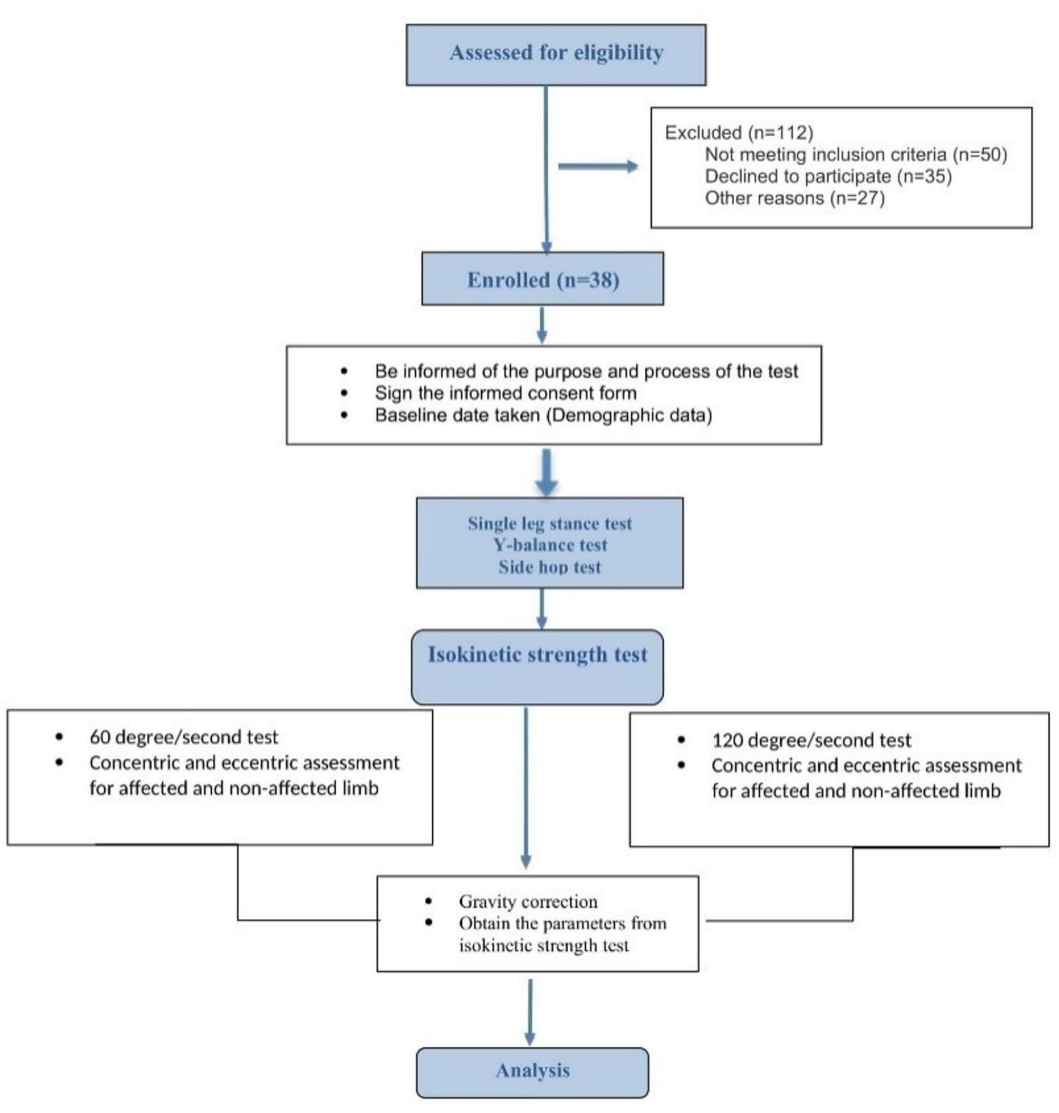
Flow chart of the study

The primary measure in this study was gastrocnemius and soleus muscles strength. Static, dynamic balance and functional performance were secondary outcome measures. The steps of the testing protocol are shown in Fig. 1.

To minimize bias and assure reliability, all participants were evaluated under similar settings and following the same standardized procedure with the same researcher, with consistently given instructions and outcome measurements recorded accurately. Besides, the researcher conducting the strength and functional tests assessment was blinded to the participants’ IdFAI scores and ankle instability status.

### Gastrocnemius and soleus muscle strength assessment

Gastrocnemius and soleus muscles strength were measured using a Cybex isokinetic dynamometer (model HUMAC NORM, Cybex International, Inc., Ronkonkoma, NY). It is a valid tool in measuring muscle strength and has intraclass correlation values ranging from 0.81 to 0.99, which indicates ‘very good’ to ‘excellent ‘reliability [31]. Before testing, participants underwent a standardized warm-up protocol to prepare the musculature and reduce injury risk. This warm-up involved 5 minutes of stationary cycling at a self-selected moderate intensity, followed by a series of light static and dynamic stretches focusing on the gastrocnemius and soleus muscles.

**Fig. 2:**
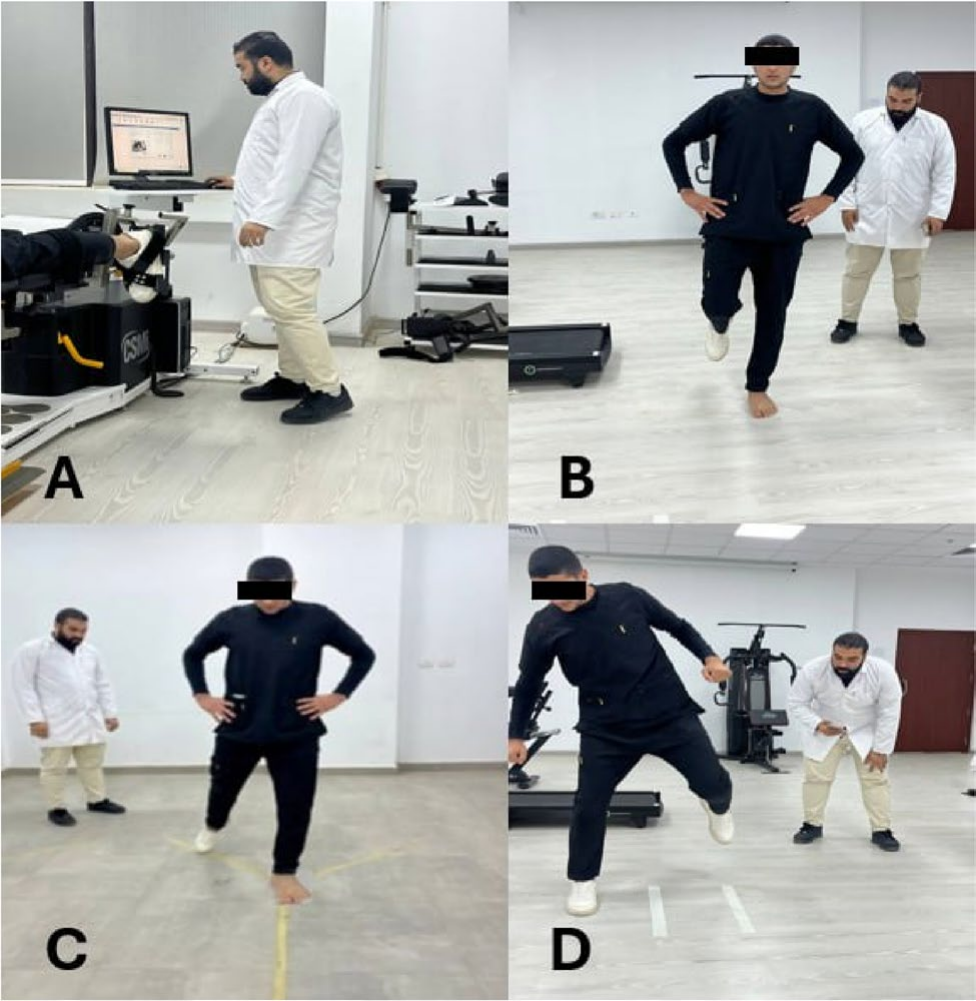
(**A**) Isokinetic testing of gastrocnemius and soleus muscles Strength, (**B**) Single leg stance (Eyes open/closed), (**C**) Y-balance test, (**D**) Side hop test

To start the test, the participant was positioned prone on the dynamometer chair with their knee fully extended (0° flexion) and their ankle in a neutral position (0° dorsiflexion/plantar flexion). The dynamometer’s axis of rotation was positioned with the lateral malleolus of the ankle. The foot was securely fastened to the dynamometer’s footplate using adjustable straps, ensuring no movement between the foot and the attachment. Additional straps were placed across the thigh and lower back to stabilize the body and isolate the ankle joint movement. Participants received clear instructions on the testing procedure and were allowed to perform several submaximal practice repetitions to familiarize themselves with the required movement and resistance. The testing protocol consisted of two sets of five maximal repetitions, each for concentric and eccentric plantarflexion at angular velocities of 60°/s and 120°/s. The order of testing (concentric vs. eccentric at 60°/s and 120°/s) was randomized. A 60-second rest period was provided between each set to minimize fatigue [32]. During each repetition, participants were instructed to push their foot down (plantarflexion) as hard and as fast as possible through the full range of motion (from 10° dorsiflexion to 30° plantarflexion). Verbal encouragement was provided consistently throughout the testing to ensure maximal effort. The dynamometer’s computer software recorded torque curves for each repetition. The highest peak torque value from the five repetitions for each condition (concentric 60°/s, concentric 120°/s, eccentric 60°/s, eccentric 120°/s) was identified and used for analysis. These values were then normalized to body weight (Nm/kg) and multiplied it by 100% to account for differences in body size among participants. Both the affected and non-affected limbs were evaluated using identical procedures, with the order of limb testing randomized (Fig. 2.A) [33].

### Static balance assessment

The SLS test was employed as a valid tool to assess static balance control, which is often impaired in individuals with functional ankle instability [34]. It has an interrater reliability of 0.898 [35]. The test was conducted on a firm, level surface. A digital stopwatch (Accusplit Pro Survivor A601X, Accusplit, Inc., Livermore, CA) with 0.01-second precision was used for timing. For the testing, participants were barefoot and wore comfortable clothing. They were given verbal instructions and a demonstration of the proper stance technique. The test was performed under two conditions: eyes open and eyes closed. For the eyes-open condition, a visual target was placed at eye level on a wall three meters in front of the participants. They were instructed to stand on their tested leg, ensuring that their foot was centered on the ground. Then lift their non-tested foot off the ground by flexing their non-tested hip and knee to 90 degrees so that the thigh was parallel to the ground and the foot was raised clear of the ground with their hands on their hips. Lastly, they were asked to maintain this position as much as they could with eyes opened. For the eyes-closed condition, participants assumed the same position and then closed their eyes. The researcher visually monitored the lifted leg during testing. A timer started counting when they lifted their non-tested foot off the ground. Then, the timer stopped when any of the following occurred: the non-tested foot touched the ground, the stance foot moved, hands were removed from hips, eyes opened (Fig. 2.B). Three trials were conducted for each condition (eyes open and closed) on both the affected and non-affected limbs, with a 30-second rest between trials. Both the limb and visual condition tests were administered in randomized order. The duration of each trial was recorded to the nearest 0.01 s. The best (longest) time out of the three trials for each condition and limb was used for analysis [36].

### Dynamic balance assessment

The YBT is a valid test to assess dynamic balance and neuromuscular control. It has been widely used in assessing individuals with functional ankle instability with excellent intra-rater reliability (Intraclass Correlation Coefficients (ICCs) = 0.88–0.99) [37]. The YBT Kit (Functional Movement Systems, Chatham, VA) was used for standardized testing. The kit consists of a stance platform and three polyvinyl chloride (PVC) pipes attached to the anterior, posteromedial, and posterolateral directions, forming a Y shape. The pipes were marked in 5 mm increments for precise measurement. Participants were barefooted and wore tight-fitting shorts to visualize anatomical landmarks. Before testing, leg length was measured from the anterior superior iliac spine to the distal tip of the medial malleolus while the participants lay supine for score normalization. They stood on the center platform with their toes behind the starting line. They were instructed to maintain a single-leg stance while reaching as far as possible with the free limb in each of the three directions (anterior, posteromedial, and posterolateral), gently pushing the reach indicator along the pipe (Fig. 2.C). Six practice trials were performed in each direction, followed by three recorded trials. The order of testing directions was randomized, but all three trials in one direction were completed before moving to the next direction [38]. The maximal reach distance was recorded to the nearest 0.5 cm for each direction. The composite score was calculated by dividing the sum of reach distances in three directions by three times the limb length and then multiplied by 100 to get it as a percentage [38].The six familiarization trials based on prior research indicating that 4–6 trials are sufficient to minimize learning effects without fatigue, and participants were given brief rest intervals to account for fatigue without complaints [37, 38].

### Functional performance test

The side hop test (SHT) is a valid tool to assess lateral stability, power, and neuromuscular control of the ankle joint complex. The interrater ICC ranged from 0.83 to 0.91. It was chosen for its relevance to the quick lateral movements often associated with ankle instability episodes and its established reliability in assessing individuals with FAI [26, 39]. Two parallel lines were marked on a non-slip floor surface, 30 cm apart, using athletic tape. The testing area was cleared of obstacles within a 2-meter radius for safety, and a digital stopwatch with 0.01-second precision was used for timing. Before the test, participants engaged in a brief warm-up that comprised 2 min of light jogging, followed by dynamic stretching of the lower limbs. Then, they received a visual demonstration and verbal instructions to ensure that they were familiar with the proper way for the SHT. Participants began the test by standing on one leg (the tested leg) with their foot aligned next to one of the taped lines (Fig. 2.D). They were instructed to hop laterally over the second line and then immediately hop back over the first line. This constituted one complete repetition. Participants were required to perform 10 complete repetitions as quickly as possible while maintaining balance and control. The timer started when the participant’s foot left the ground for the first hop and stopped when they landed after the 10th repetition. Participants were instructed to keep their hands on their hips throughout the test to standardize upper body movement and ensure focus on lower limb performance [40].

Three trials were conducted on each leg, with a 45-second rest period between trials to minimize fatigue effects. The order of testing (affected vs. non-affected limb) was randomized for each participant. The time for each trial was recorded to the nearest 0.01 s. The best (fastest) time out of the three trials for each leg was used for analysis. Additionally, the number of balance errors (touching the ground with the non-test foot, removing hands from the hips) was recorded for each trial. A trial was considered invalid and repeated if participants failed to hop the full distance, performed incorrect repetitions, or removed their hands from their hips for more than a momentary loss of balance [41]. All assessments were conducted by the same experienced and blind researcher. For all participants, the functional tests (SLS, YBT, SHT) were conducted before isokinetic measurements of gastrocnemius and soleus muscle strength to eliminate fatigue-related disturbances in balance [42], with overall 20-minutes rest period between each assessment [43].

### Statistical analysis

The G*Power program (version 3.1.9.7) was employed to perform a priori power analysis for a two-tailed independent t-test. The computation used plantar flexor torque divided by body weight, with α set to 0.05 and power (1 - β err prob) at 80%. The effect size, Cohen’s d = 0.698, is based on previously published study [19]. A total of 38 participants were required for the study. All statistical analyses were conducted using Python, applying pandas for data handling, SciPy for tests, and stats models for regression. Descriptive statistics were calculated for demographic data (meaning SD, range, and proportions). The Shapiro-Wilk test was used to assess the normality of strength data, which then guided the use of paired t-tests to compare gastrocnemius and soleus muscles strength between affected and non-affected limbs, with effect sizes (Cohen’s d) for each comparison. A two-way ANOVA assessed the main effects of limb and velocity on strength, including their interaction, with effect size (partial η²). Correlation coefficients (r) were interpreted according to Cohen’s benchmarks: small (0.10–0.29), moderate (0.30–0.49), and large (≥ 0.50) [44]. Alternative classification scheme provided by Evans uses similar thresholds [45]. Pearson correlations evaluated the relationship between strength deficits and functional outcomes. Multiple linear regression analyses were conducted to explore the impact of strength deficits on functional outcomes, with significant results illustrated in scatter plots with regression lines. Statistical significance was set at *p* < 0.05.

## Results

The study included 38 participants (28 males, 10 females) with mean age 21.2 ± 2.2 years and BMI 23.6 ± 1.7 kg/m². Right limb instability was more prevalent (73.7%) (Table 1).

**Table 1:** Study characteristics of included participants

**Demographic Variable**	**Mean ± SD [Range]**
Age (years)	21.2 ± 2.2 [17.0–26.0]
Weight (Kg)	70.9 ± 9.9 [50.0–87.0]
Height (Cm)	173.1 ± 8.7 [155.0–190.0]
BMI (kg/m²)	23.6 ± 1.7 [19.5–27.9]
Sex	Percent (%)
Male	73.7%
Female	26.3%
Affected Limb	Percent (%)
RT	73.7%
LT	26.3%
IdFAI	Mean ± SD
Affected	15.61 ± 2.79
Non-affected	5.42 ± 3.04

*Abbreviation* *SD* standard deviation, *BMI* Body mass index, *IdFAI* Identification of functional ankle instability, *%* percent, *LT* Left, *RT* Right

A two-way ANOVA revealed a significant main effect of limb (F 1, 37) = 26.99, *p* < 0.001, Partial η² = 0.42), but no significant main effect of velocity (*p* = 0.505) or interaction between limb and velocity (*p* = 0.859) (Table 2).

**Table 2:** Two –way ANOVA for main effects of limb and velocity on gastrocnemius and soleus muscles strength

Effect	F Value	df (Num, Den)	*p*-Value	Partialη²
Main Effect of Limb	26.99	1, 37	< 0.001*	0.42
Main Effect of Velocity	0.45	1, 37	0.505	0.01
Interaction (Limb × Velocity)	0.03	1, 37	0.859	0.001

Footnotes: Partial η² = Effect size; Small (0.01), Medium (0.06), Large (0.14)

^*^ Indicates significant difference (*p* < 0.05), df degree of freedom, Num numerator, Den denominator

### Comparison of gastrocnemius and soleus muscles strength, balance, and functional performance between affected and non-affected limb

**Fig. 3:**
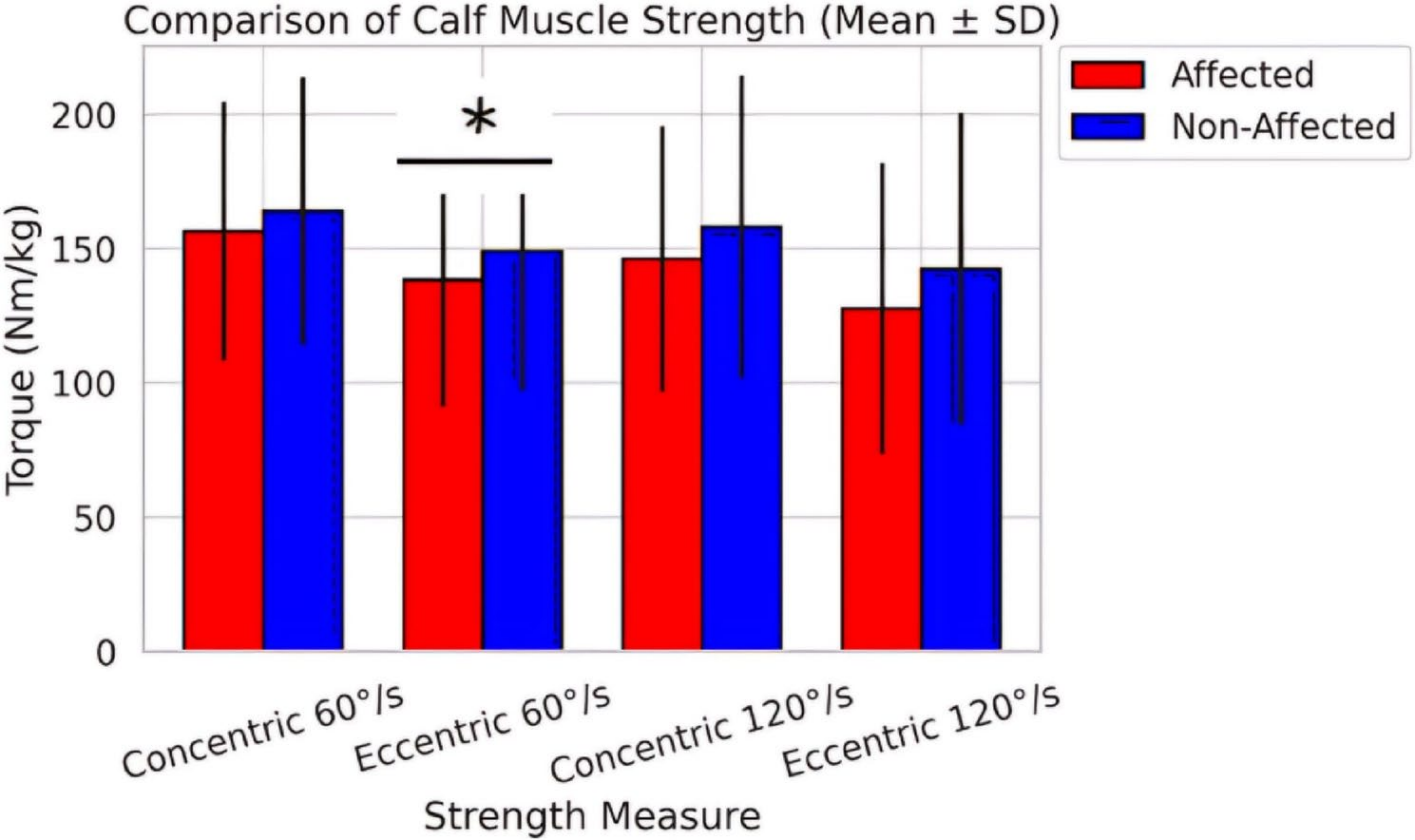
Bar chart with error bars comparing calf muscle strength (concentric and eccentric at 60°/s and 120°/s) between affected and non-affected limbs; *means significance differences (*P*<0.05)

Regarding muscle strength, paired t-tests revealed a significant difference between affected and non-affected limbs only for eccentric torque at 60°/s (*p* = 0.048), with the affected limb showing lower strength (127.36 ± 42.10 Nm/kg vs. 149.10 ± 51.91 Nm/kg) (Fig. 3), while there were significant differences in all balance and functional performance between affected and non-affected limbs (*p*<0.05). The static balance with eyes open and closed, and the dynamic balance all showed worse performance in the affected limb (*p* = 0.044, *p* = 0.004, and *p*< 0.001, respectively). The affected limb revealed poorer functional performance than the non-affected limb (*p* = 0.036) (Table 3).

**Table 3:** Paired t-test comparisons of gastrocnemius and soleus muscles strength, balance, and functional performance between affected and non-affected limbs

Variable	Mean ± SD (Affected)	Mean ± SD (Non-Affected)	t-Value	*p*-Value	Effect Size (Cohen’s d)
Concentric Torque (60°/s) (BW%)	156.51 ± 48.21	163.98 ± 49.78	−1.19	0.241	−0.15
Eccentric Torque (60°/s) (BW%)	127.36 ± 42.10	149.10 ± 51.91	−2.1	0.048*	−0.46
Concentric Torque (120°/s) (BW%)	146.11 ± 49.54	158.20 ± 56.47	−1.51	0.139	−0.23
Eccentric Torque (120°/s) (BW%)	127.76 ± 54.29	142.43 ± 58.15	−1.34	0.189	−0.26
Static balance (Eyes Open) (Sec)	56.40 ± 20.56	64.55 ± 24.11	2.08	0.044*	0.36
Static balance (Eyes Closed) (Sec)	7.63 ± 1.99	9.21 ± 2.33	3.11	0.004*	0.73
Dynamic balance (%)	63.32 ± 9.10	71.39 ± 7.68	6.48	< 0.001*	0.96
Functional performance (Sec)	18.08 ± 4.14	16.28 ± 3.14	2.13	0.036*	−0.49

Footnotes, body weight%; Effect size [Cohen’s d), Magnitude of difference between limbs, with interpretation as follows: Small [|d| <0.3), Medium [|d| < 0.8), Large [|d| ≥ 0.8); t-Value, Test statistic from the paired t-test comparing affected and non-affected limbs for each outcome measure

^*^ Indicates significant difference (*p* < 0.05)

*Abbreviations* *Mean ± SD* Mean value ± standard deviation, *Sec* seconds, *%* percent, *(BW%)* percentile body weight

### Relationship between gastrocnemius and soleus muscles strength, balance, and functional performance in the affected limb 

**Fig. 4:**
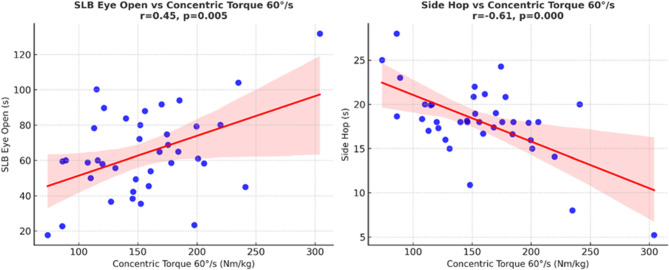
Scatter plot with regression line showing the relationship between Static Balance (SLS) Eye Open (s) and Concentric Torque 60°/s (BW%) in the affected limb group. Scatter plot with regression line showing the relationship between Functional performance (SHT)(s) and Concentric Torque 60°/s (BW % ) in the affected limb group

As shown in Table 4, static balance with eyes open demonstrated moderate positive correlations with concentric torque at both 60°/s (r = 0.45, *p* <.001) and 120°/s (r = 0.46, *p* <.001). A small correlation was also observed with eccentric torque at 120°/s (r= 0.29,* p* =.08), although this did not reach statistical significance. Dynamic balance showed moderate positive correlations with concentric torque at 60°/s (r = 0.41, *p* =.01) and 120°/s (r = 0.34, *p* =.04), and with eccentric torque at 60°/s (r = 0.35, *p* =.03). A small, non-significant correlation was observed with eccentric torque at 120°/s (r = 0.27, *p* =.10). Functional performance demonstrated moderate-to-strong negative correlations with all plantar flexors muscle strength measures, with coefficients ranging from r= −0.49 to r = −0.61 (all *p* <.001). These findings suggest that greater gastrocnemius and soleus muscles strength is associated with shorter side hop times, reflecting superior functional performance (Fig. 4).

**Table 4:** Correlation between gastrocnemius and soleus muscles strength, balance, and functional performance in the affected group

Functional outcome variables	Gastrocnemius and soleus muscles strength	Correlation (*r*)	*p*-Value
Static balance [Eyes Open] (Sec)	Concentric Torque [60°/s] (BW%)	0.45	< 0.001 ^*^
	Eccentric Torque [60°/s] (BW%)	0.32	0.05
	Concentric Torque [120°/s] (BW%)	0.46	< 0.001*
	Eccentric Torque [120°/s] (BW%)	0.29	0.08
Static balance [Eyes Closed] (Sec)	Concentric Torque [60°/s] (BW%))	0.15	0.36
	Eccentric Torque [60°/s] (BW%)	0.12	0.46
	Concentric Torque [120°/s] (BW%)	0.2	0.24
	Eccentric Torque [120°/s] (BW%)	0.14	0.4
Dynamic balance (%)	Concentric Torque [60°/s] (BW%)	0.41	0.01*
	Eccentric Torque [60°/s] (BW%)	0.35	0.03*
	Concentric Torque [120°/s] (BW%)	0.34	0.04*
	Eccentric Torque [120°/s] (BW%)	0.27	0.1
Functional performance (Sec)	Concentric Torque [60°/s] (BW%)	−0.61	< 0.001*
	Eccentric Torque [60°/s] (BW%)	−0.53	< 0.001*
	Concentric Torque [120°/s] (BW%)	−0.49	< 0.001*
	Eccentric Torque [120°/s] (BW%)	−0.51	< 0.001*

Footnotes: Correlation interpretation: Weak (0.1–0.3), Moderate [0.3–0.5), Strong [> 0.5)

^*^ Indicates significant difference (*p* < 0.05)

*Abbreviations* *%*, Percentage, *r* correlation coefficient, *BW%* percentile body weight, *Sec* Second

Multiple regression analyses examined the relationship between strength deficits and functional outcomes. The static balance (Eyes Open) model explained 14.8% of the variance (Adjusted R² = 0.044), with no significant predictors. The static balance (Eyes Closed) model explained 41.7% of the variance (Adjusted R² = 0.346), with significant predictors including eccentric torque deficit at 60°/s (β = 0.04, *p* = 0.018), concentric torque deficit at 120°/s (β = 0.074, *p* = 0.003), and eccentric torque deficit at 120°/s (β = −0.057, *p* < 0.001). The dynamic balance model explained 19.8% of the variance [Adjusted R² = 0.101) and the functional performance model explained 33.6% of the variance (Adjusted R² = 0.256), both with no statistically significant predictors (Table 5). The full regression equations, including intercepts, unstandardized coefficients, 95% confidence intervals, and model fit indices, are provided in Appendix A for completeness

**Table 5:** Regression analyses predicting functional outcomes from strength deficits (only variables showing significant correlation were included)

Outcome (Unit)	Predictor (Δ, (BW%))	B	SE	95% CI	t	*p*	*R*²	Adj. *R*²	Tolerance	VIF
Static balance [Eyes Open] (Sec)	Concentric Torque 60°/s	−0.244	0.329	[−0.912, 0.425]	−0.74	0.463	0.148	0.044	0.053	18.884
	Concentric Torque 120°/s	0.553	0.341	[−0.140, 1.246]	1.62	0.114			0.102	9.792
Dynamic balance (%)	Concentric Torque 60°/s	−0.105	0.094	[−0.297, 0.087]	−1.11	0.276	0.198	0.101	0.102	9.792
	Eccentric Torque 60°/s	0.090	0.067	[−0.047, 0.226]	1.34	0.190			0.053	18.884
	Concentric Torque 120°/s	0.051	0.098	[−0.148, 0.250]	0.53	0.603			0.102	9.828
Functional performance (Sec)	Concentric Torque 60°/s	−0.087	0.050	[−0.188, 0.014]	−1.76	0.088	0.336	0.256	0.100	10.040
	Eccentric Torque 60°/s	0.012	0.035	[−0.060, 0.084]	0.33	0.741			0.052	19.337
	Concentric Torque 120°/s	−0.021	0.052	[−0.125, 0.084]	−0.40	0.693			0.052	19.232
	Eccentric Torque 120°/s	0.029	0.032	[−0.037, 0.094]	0.90	0.376			0.108	9.293

Footnote: Δ = difference between affected and non-affected limbs (strength deficit, measured in BW%); B = unstandardized regression coefficient; SE = standard error of the coefficient; 95% CI = 95% confidence interval; t = t-statistic for the coefficient; p = p-value indicating significance of the predictor; R² = coefficient of determination, proportion of variance explained by the model; Adj. R² = adjusted R², coefficient of determination adjusted for number of predictors. VIF = Variance Inflation Factor, Values >10 (or tolerance <0.10) were considered indicative of problematic collinearity. *p < 0.05 considered statistically significant

*Abbreviations* *%*, Percentage, BW % body weight percentile, *r* correlation coefficient, *Sec* Seconds, *Std* standard deviation, VIF Variance inflation factor

## Discussion

This study aimed to explore various aspects of gastrocnemius and soleus muscles strength and its consequences in participants with FAI, specifically; it compared the concentric and eccentric gastrocnemius and soleus muscles strength between the affected limb with instability and the non-affected limb. Furthermore, this study examined how deficits in gastrocnemius and soleus muscles strength might be predictors for alterations of the three functional outcomes: static and dynamic balance, as well as functional performance in participants with FAI.

A total of thirty-eight participants with an age range (17.0–26.0.0years) and a BMI range from 19.5 to 27.9 kg/m², with 73.7% had FAI in the right ankle and 26.3% had instability in the left ankle. This distribution reveals limb dominance in functional movement patterns, which may contribute to the development of instability in one limb over the other.

The results reported no significant differences in gastrocnemius and soleus muscles strength between the affected and non-affected limbs across different contraction velocities (60°/s and 120°/s) for both concentric and eccentric movements, with only significant difference of eccentric torque at 60°/s (*p* = 0.048) with a moderate effect size. This finding is noteworthy as eccentric ankle control is crucial for delaying tibial advancement and maintaining balance during activities like landing and directional changes. Diminished strength at slower velocities may indicate sensorimotor abnormalities in FAI patients, explaining instability and emphasizing the importance of including eccentric strengthening in rehabilitation treatments. This aligns with prior findings that emphasize the significance of eccentric strength in maintaining ankle stability, especially at lower angular velocities where controlled deceleration and force absorption are necessary for participants with FAI [19].

The study concluded that contraction velocity had no effect on muscle strength, with a substantial main effect of limb and no significant effects of velocity or limb-velocity interaction. These findings are explained by the peak torque normally remains constant at low-to-moderate speeds and primarily decreases at higher angular velocities [46]. On the other hand, the significant limb main effect signifies the persistent strength differences between the unstable and stable ankles. Additionally, the strength deficit remains across a variety of contraction velocities comes from the lack of a limb × velocity interaction. This highlights that FAI is linked to general muscle weakness in the affected limb regardless of speed, suggesting rehabilitation strategies should focus on overall strength restoration [47].

This study’s findings revealed moderate to strong direct relationships between gastrocnemius and soleus muscles strength and balance measures, particularly in dynamic balance and functional performance. These might be due to the substantial role of ankle plantar flexors muscles in maintaining ankle stability, postural stability during locomotion and functional activities [20, 22]. This aligns with previous research that signify that higher muscle strength is linked to better dynamic stability and movement control around the ankle joint [24].

The functional performance demonstrated strong indirect correlations with both concentric and eccentric torque, indicating that participants with weaker gastrocnemius and soleus muscles performed worse in agility tasks. This is explained as the ankle instability is associated with reduced muscle strength, reinforcing the link between strength asymmetries and functional movement impairments [48].

The multiple regression analysis examined the predictive value of gastrocnemius and soleus muscles strength deficits on functional outcomes. The static balance (Eyes Open) performance was not significantly affected by strength deficits, suggesting that muscle asymmetry alone does not determine balance stability under open-eye conditions. However, static balance (Eyes Closed) performance was significantly influenced by strength deficits, with eccentric torque deficit at 60°/, concentric torque deficit at 120°/s, and eccentric torque deficit at 120°/s emerging as key predictors. This may indicate greater strength deficits, particularly in eccentric contractions, contribute to poorer balance when visual feedback is limited. In contrast, functional performance and dynamic balance were not strongly predicted by strength deficits, suggesting that neuromuscular control, coordination, and other biomechanical factors play a larger role in dynamic movement efficiency.

According to multicollinearity analysis, certain measures of plantar flexors muscle strength had high VIFs, suggesting overlap between predictors. This overlap might be due to the probability of coexisting relationship between independent variables hindering some variables to show significance in the regression model. The distinct contributions of gastrocnemius and soleus muscle strength components to balance and functional outcomes in patients with FAI may become more apparent with the use of larger sample sizes in future studies.

These findings highlight that strength deficits, especially in eccentric contractions, are critical for static balance control (Eyes Closed) but have a limited impact on dynamic functional tasks like SHT and YBT [49]. It highlights the role of these deficits in impairing balance and functional performance, offering targeted insights for rehabilitation.

In contrast to the findings of this study, a previous study focused primarily on the concentric strengthening of gastrocnemius and soleus muscles as the main factor responsible for improving stability, balance, and reducing fall risk in older adults [50], the current study engaged a dual-velocity approach, consistent with the findings of Singh et al. [51], who also employed a dual-velocity testing strategy. This method is crucial for accurately assessing muscle conditions and provides a more effective means of tracking functional abnormalities. Additionally, it ascertains the significant role of the plantar flexor muscles in maintaining optimal function during different activities.

The results of the current study support the findings of Rosen et al. [52] who found that proprioceptive deficits were associated with poor postural control in participants with FAI, as it strengthens the link between proprioception and functional stability by linking these deficiencies to muscle strength, specifically in eccentric contractions. Research suggests that eccentric muscle strength and proprioception are closely related as eccentric contractions provide constant sensory input from mechanoreceptors while also decelerating and stabilizing ankle joint motion. This afferent feedback may be diminished by decreased eccentric strength, which could lead to proprioceptive deficiencies and compromised dynamic and postural stability, resulting in functional ankle instability [53, 54].

This study contrasts with the findings of Abdel-Aziem and Draz [6], which examined ankle muscle strength ratios and concluded that CAI significantly altered muscle ratios by reducing the eversion/inversion ratio while increasing the dorsiflexion/plantarflexion ratio. However, their study did not isolate the plantar flexors for detailed analysis or establish a correlation between balance and functional performance.

The study of Park et al. [25] found that inversion muscle strength in the affected ankle was lower than in the non-affected one, but no significant differences were found in eversion, dorsiflexion, or plantarflexion muscle strength. The study also examined the relationship between inversion strength and functional performance using the single heel raise test, with measurements taken at angular velocities of 30°/s and 180°/s. However, the present study assessed gastrocnemius and soleus muscles strength at 60°/s and 120°/s and identified significant deficits in plantar flexor strength. These signify the importance of velocity-specific strength assessments for effective interventions based on functional demands.

Moreover, Lajevardi et al. [20] reported that concentric muscle strength, particularly in the plantar flexors (gastrocnemius and soleus), is the key determinant of ankle stability, functional mobility, and gait speed, however, the research challenges this view, arguing that eccentric muscle control plays a far more pivotal role. While concentric strength generates movement, eccentric strength stabilizes, absorbs impact, and refines control, making it the foundation for sustained ankle function and injury prevention [6, 19].

Up to date, this research provides a method for rehabilitation by examining the impact of muscle strength at specific velocities on functional performance, besides the relationship between deficits gastrocnemius and soleus muscles strength (isokinetic measurements) and functional deficits (balance measurements) in participants with FAI. The study’s dependability was enhanced by accurate torque measurements using a Cybex isokinetic dynamometer, which is non-affected by subjective bias [55]. This study reveals a correlation between gastrocnemius and soleus muscles strength deficiencies and functional tests, offering practical insights for creating tailored rehabilitation programs, emphasizing velocity-specific training for customized therapies in clinical practice. Despite the promising findings, the study had limitations that include its focus on young individuals, which may not apply to elderly or comorbid populations. It also focused only on gastrocnemius and soleus muscles strength and torque. The unequal gender distribution of this study may limit the generalization of the findings due to sex-related variations in muscular strength and functional performance. The small sample size may have hindered the detection of real correlations and increased variable, and predictor overlaps in the regression analysis. Future research should assess plantar flexors, dorsiflexors, evertors, and invertors, which significantly influence ankle stability. These muscle groups are essential for understanding the role of total lower-limb strength in FAI, which may not be fully understood without these.

Other neuromuscular tests like electromyography are needed to evaluate the activation and timing of muscle groups around the ankle that affect its stability. Future studies should assess a more balanced gender distribution to explore the potential variations in the associations between plantar flexors strength and functional performance outcomes.

## Conclusion

The study explores the link between FAI and gastrocnemius and soleus muscles strength deficits, emphasizing the importance of eccentric strength and velocity-specific testing. It found moderate associations between gastrocnemius and soleus muscles strength deficits and functional outcomes, particularly in dynamic balance and functional performance. Strength deficits significantly predicted performance in static balance (SLS with eyes closed), indicating muscle strength is linked to improved functional performance.The study provides good recommendations for FAI rehabilitation, bridging laboratory measurements with functional outcomes, contributing to our understanding of FAI, and laying the groundwork for future studies and improved clinical practices.

## Data Availability

Dataset used or analyzed during the current study are available on reasonable request from the corresponding author.

## Appendix

### Final Regression Model Equations and Tables

#### Static balance SLS Eye Open (s)

*Model fit: R² = 0.148, Adjusted R² = 0.044, n = 38, df = 33.*

**Table:**

Predictor (deficit, Nm)	B	SE	95% CI	t	p
Intercept	63.879	3.982	[55.777, 71.980]	16.04	<.001
Concentric 60°/s (Δ)	-0.244	0.329	[-0.912, 0.425]	-0.74	0.463
Eccentric 60°/s (Δ)	-0.009	0.233	[-0.484, 0.466]	-0.04	0.969
Concentric 120°/s (Δ)	0.553	0.341	[-0.140, 1.246]	1.62	0.114
Eccentric 120°/s (Δ)	-0.341	0.213	[-0.773, 0.092]	-1.6	0.119

Δ = Affected − Non-affected; outcomes in native units; predictors in Nm. Coefficients are unstandardized. 95% CIs based on two-sided 0.05 level

Equation:

$$\begin{aligned}\widehat{\mathrm Y}=& 63.879+(-0.244)\cdot\mathrm{Con}60\mathrm\Delta\\&+(-0.009)\cdot\mathrm{Ecc}60\mathrm\Delta+(0.553)\\&\cdot\mathrm{Con}120\mathrm\Delta+(-0.341)\cdot\mathrm{Ecc}120\mathrm\Delta\end{aligned}$$

#### Static balance (SLS)Eye Close (s)

*Model fit: R² = 0.417, Adjusted R² = 0.346, n = 38, df = 33.*

**Table:**

Predictor (deficit, Nm)	B	SE	95% CI	t	p
Intercept	7.879	0.271	[7.327, 8.430]	29.07	<.001
Concentric 60°/s (Δ)	-0.031	0.022	[-0.077, 0.014]	-1.4	0.171
Eccentric 60°/s (Δ)	0.040	0.016	[0.007, 0.072]	2.49	0.018
Concentric 120°/s (Δ)	0.074	0.023	[0.027, 0.121]	3.19	0.003
Eccentric 120°/s (Δ)	-0.057	0.014	[-0.086, -0.027]	-3.92	<.001

Δ = Affected − Non-affected; outcomes in native units; predictors in Nm. Coefficients are unstandardized. 95% CIs based on two-sided 0.05 level

Equation:

$$\begin{aligned}\widehat{\mathrm Y}=&7.879+(-0.031)\cdot\mathrm{Con}60\mathrm\Delta\\&+(0.040)\cdot\mathrm{Ecc}60\mathrm\Delta+(0.074)\\&\cdot\mathrm{Con}120\mathrm\Delta+(-0.057)\cdot\mathrm{Ecc}120\mathrm\Delta\end{aligned}$$

### Dynamic balance

#### Y-Balance (%)

*Model fit: R² = 0.198, Adjusted R² = 0.101, n = 38, df = 33.*

**Table:**

Predictor (deficit, Nm)	B	SE	95% CI	t	p
Intercept	70.337	1.143	[68.011, 72.664]	61.52	<.001
Concentric 60°/s (Δ)	-0.105	0.094	[-0.297, 0.087]	-1.11	0.276
Eccentric 60°/s (Δ)	0.090	0.067	[-0.047, 0.226]	1.34	0.190
Concentric 120°/s (Δ)	0.051	0.098	[-0.148, 0.250]	0.53	0.603
Eccentric 120°/s (Δ)	-0.087	0.061	[-0.212, 0.037]	-1.43	0.162

Δ = Affected − Non-affected; outcomes in native units; predictors in Nm. Coefficients are unstandardized. 95% CIs based on two-sided 0.05 level

Equation:

$$\begin{aligned}\widehat Y=&70.337+(-0.105)\cdot Con60\Delta\\&+(0.090)\cdot Ecc60\Delta+(0.051)\\&\cdot Con120\Delta+(-0.087)\cdot Ecc120\Delta\end{aligned}$$

#### Functional performance Side Hop (s)

*Model fit: R² = 0.336, Adjusted R² = 0.256, n = 38, df = 33*

**Table:**

Predictor (deficit, Nm)	B	SE	95% CI	t	p
Intercept	17.729	0.602	[16.504, 18.955]	29.44	<.001
Concentric 60°/s (Δ)	-0.087	0.050	[-0.188, 0.014]	-1.76	0.088
Eccentric 60°/s (Δ)	0.012	0.035	[-0.060, 0.084]	0.33	0.741
Concentric 120°/s (Δ)	-0.021	0.052	[-0.125, 0.084]	-0.40	0.693
Eccentric 120°/s (Δ)	0.029	0.032	[-0.037, 0.094]	0.90	0.376

Δ = Affected − Non-affected; outcomes in native units; predictors in Nm. Coefficients are unstandardized. 95% CIs based on two-sided 0.05 level

Equation:

$$\begin{aligned}\widehat Y=&17.729+(-0.087)\cdot Con60\Delta\\&+(0.012)\cdot Ecc60\Delta+(-0.021)\\&\cdot Con120\Delta+(0.029)\cdot Ecc120\Delta\end{aligned}$$

## Abbreviations

FAI: Functional ankle instability
SLS: Single-leg stance
SHT: Side hop test
YBT: Y-balance test
